# Long-range interdomain communications in eIF5B regulate GTP hydrolysis and translation initiation

**DOI:** 10.1073/pnas.1916436117

**Published:** 2020-01-03

**Authors:** Bridget Y. Huang, Israel S. Fernández

**Affiliations:** ^a^Department of Biochemistry and Molecular Biophysics, Columbia University, New York, NY 10032

**Keywords:** translation, ribosome, initiation, eIF5B

## Abstract

Translation initiation controls protein synthesis by regulating the delivery of the first aminoacyl-tRNA to messenger RNAs (mRNAs). In eukaryotes, initiation is sophisticated, requiring dozens of protein factors and 2 GTP-regulated steps. The GTPase eIF5B gates progression to elongation during the second GTP-regulated step. Using electron cryomicroscopy (cryo-EM), we imaged an in vitro initiation reaction which is set up with purified yeast components and designed to stall with eIF5B and a nonhydrolyzable GTP analog. A high-resolution reconstruction of a “dead-end” intermediate at 3.6 Å allowed us to visualize eIF5B in its ribosome-bound conformation. We identified a stretch of residues in eIF5B, located close to the γ-phosphate of GTP and centered around the universally conserved tyrosine 837 (*Saccharomyces cerevisiae* numbering), that contacts the catalytic histidine of eIF5B (H480). Site-directed mutagenesis confirmed the essential role that these residues play in regulating ribosome binding, GTP hydrolysis, and translation initiation both in vitro and in vivo. Our results illustrate how eIF5B transmits the presence of a properly delivered initiator aminoacyl-tRNA at the P site to the distant GTPase center through interdomain communications and underscore the importance of the multidomain architecture in translation factors to sense and communicate ribosomal states.

## Significance

Translation is a key regulatory step in the control of gene expression. The first stage of translation, initiation, establishes the foundation for the sequential synthesis of a protein. In eukaryotes, 2 GTP-regulated checkpoints ensure the efficiency and fidelity of translation initiation. The GTPase eIF5B is responsible for the correct functioning of the second checkpoint. Molecular interactions of eIF5B with other correctly assembled components on the ribosome lead to GTP hydrolysis that allows the machinery of protein production to transition from initiation into elongation. Here, we show how a highly conserved stretch of residues in eIF5B, identified using electron cryomicroscopy, coordinates the gating into elongation, underscoring the importance of modular architecture in translation factors to sense and communicate ribosomal states.

Translation in higher eukaryotes is a complex endeavor (1). Eukaryotic ribosomes not only have to synthetize proteins, but they are required to do so in a strictly regulated fashion by delivering proteins in specific cellular contexts in space and time (2). In order to meet these demands, protein biosynthesis in eukaryotes involves dozens of protein factors that not only assist ribosomes in basic ribosomal functions like aminoacyl-tRNA delivery or translocation, but also assist in regulatory tasks that are specific to eukaryotic protein production (1). Many auxiliary factors assisting the eukaryotic ribosome are proteins of high complexity which exhibit multidomain architectures (3, 4). Some of these proteins are also integrated into massive protein complexes, like the initiation factor eIF3 (5, 6). The expanded eukaryotic complexity contrasts with the simplicity of the bacterial translational apparatus (7).

The bulk of eukaryotic translation regulation is implemented at the first phase of translation, known as initiation (8). In eukaryotes, this phase is very sophisticated and is initiated by the establishment of the 43S Preinitiation Complex (43S-PIC), where the small (40S) ribosomal subunit recruits initiation factors eIF1/1A/3 and 5 and a specific initiator aminoacyl-tRNA (Met-tRNA_i_^Met^) in the form of a ternary complex (TC) with eIF2 and GTP (9). The 43S-PIC is competent for messenger RNA (mRNA) recruitment via initiation factors of the eIF4 family (eIF4A/G/H and E). The 48S complex, formed after mRNA recruitment by the 43S-PIC, dynamically scans the mRNA from the 5′ end for an AUG codon in a favorable context (10, 11). The recognition of a proper AUG codon at the P site of the 40S subunit triggers a global conformational change from an open, scanning-competent configuration of the 40S to a closed, scanning-arrested state with Met-tRNA_i_^Met^ base-paired with the AUG codon (12). This conformational change is accompanied by phosphate release from eIF2 and its subsequent release from the 40S subunit together with eIF1 (13). A second GTP-regulated step is then required for the recruitment of the large (60S) ribosomal subunit (14). This is catalyzed by another initiation factor, eIF5B (15). Once a full (80S) ribosome is assembled with a proper AUG codon at the P site that is base-paired with Met-tRNA_i_^Met^, the ribosome is competent for elongation (16). GTP hydrolysis by eIF5B and its concomitant release from the ribosome, along with the remaining initiation factors, mark the end of initiation, which allows the transition into the fast, less regulated elongated phase (15).

The protein factor responsible for gating progression toward elongation, eIF5B, was originally identified in yeast by its sequence homology to bacterial initiation factor IF2, and later on, it was also identified in mammals (17). Extensive genetic, biochemical, and structural studies have allowed a detailed understanding of the GTP-dependent mechanism of the large subunit recruitment mediated by eIF5B (18, 19). The protein consists of 4 domains: a GTP-binding domain or G-domain, domain II, domain III, and domain IV (18). The G-domain and domain II are highly homologous to their corresponding domains of other GTPases involved in translation, such as bacterial EF-Tu/EF-G or their eukaryotic counterparts eEF1A/eEF2. Domains III and IV are linked by a long α-helix (α-helix 12, h12) whose integrity is required for proper function in vivo (20).

The G-domain and domain II of eIF5B are functionally associated and are responsible for the bulk of the binding to the ribosome and the GTP/GDP regulation (18, 19, 21). Conversely, domain III, domain IV, and the linker helix h12 are responsible for Met-tRNA_i_^Met^ engagement (22). The current model for how eIF5B works involves a “domain release” mechanism, which is induced by an initial, off-ribosome GTP-binding event (19). Upon GTP binding, domains III and IV gain flexibility as they are “released” from a rigid, GDP-bound conformation. Key elements of the G-domain are also structured upon GTP binding, which allows ribosome binding of eIF5B in the GTP form (21). The first eIF5B-ribosome association occurs in the context of the 40S: immediately after eIF2/eIF1 are released from the 48S complex, eIF5B interacts via its domain II with the 40S, and the flexible unit formed by domains III and IV “senses” the presence of Met-tRNA_i_^Met^ at the P site (23). If domain IV is stabilized due to proper recognition of the initiator aminoacyl-tRNA, 60S recruitment will proceed via the increased 60S/40S-interacting surface due to the presence of eIF5B (15, 21).

The large subunit is docked to the 40S subunit in the presence of P-site Met-tRNA_i_^Met^, eIF5, eIF1A, eIF3, and GTP-bound eIF5B (24). Correct positioning of the Met-tRNA_i_^Met^ upon 60S subunit recruitment promotes GTP hydrolysis by eIF5B. Specific elements of the 60S ribosomal RNA (rRNA), located at the Sarcin Ricin Loop (SRL), align a universally conserved histidine residue in the G-domain of eIF5B closer to the γ-phosphate of the GTP molecule, thereby promoting hydrolysis (21, 25). eIF5B then leaves the ribosome due to its reduced affinity for the ribosome in its GDP form.

Recent studies employing single-molecule FRET experiments with *Saccharomyces cerevisiae* components revealed a long residence time of eIF5B on the 80S complex prior to GTP hydrolysis (26). The study suggested the idea that structural rearrangements within the 80S Initiation Complex (80S-IC) were essential to induce GTP hydrolysis and the subsequent dissociation of eIF5B. Once the A site is available, elongation can proceed by the delivery of the second aminoacyl-tRNA by eEF1A and GTP (27).

Since its discovery, a second function of eIF5B, beyond large subunit recruitment and related to start-codon selection accuracy, was suggested (17, 23). A yeast strain lacking the gene coding for eIF5B was unable to derepress the starvation transcription factor GCN4. The 5′-Untranslated Region (5′-UTR) of the GCN4 gene contains 4 upstream AUG (uAUG) codons (28). In nonstarvation conditions, the TC (eIF2/Met-tRNA_i_^Met^/GTP) complex recognizes these uAUG codons as bona fide start codons, thereby preventing the expression of GCN4 from the canonical AUG codon. Leaky scanning from uAUG codons is prevented by eIF5B and only in starvation conditions, when eIF2 is phosphorylated and cannot be employed for Met-tRNA_i_^Met^ delivery, uAUG codons are bypassed, which results in recognition of the canonical AUG and the expression of GCN4. It is well established that deregulation and increase in leaky scanning is associated with the absence or mutations of eIF5B (14). Therefore, eIF5B seems to play an essential role in canonical initiation (23) by promoting efficient and accurate loading of Met-tRNA_i_^Met^ as well as in noncanonical conditions when phosphorylation of eIF2 prevents regular delivery of Met-tRNA_i_^Met^ (29). These important roles place eIF5B as a key regulator of protein synthesis. Recent reports link eIF5B expression with developmental transitions (30), regulation of translation programs under stress conditions (31), and cancer (32).

Much is known about the ribosome-binding determinants of eIF5B (21) as well as how the Met-tRNA_i_^Met^ is recognized (22). However, less is known regarding the global behavior of the protein and how ribosome binding and initiator aminoacyl-tRNA recognition activities are integrated into a unified response in activating GTP hydrolysis. Using a high-resolution electron cryomicroscopy (cryo-EM) reconstruction of an 80S/eIF5B intermediate, we identified a highly conserved group of residues that is outside of the G-domain and strategically positioned at the junction between 3 of the 4 domains of eIF5B in its ribosome-bound conformation. This cluster of residues is able to regulate GTP hydrolysis and ribosome binding, highlighting the importance of intradomain communications within eIF5B to regulate gating to elongation.

## Results

### In Vitro Initiation Reaction and Cryo-EM.

**Fig. 1.**
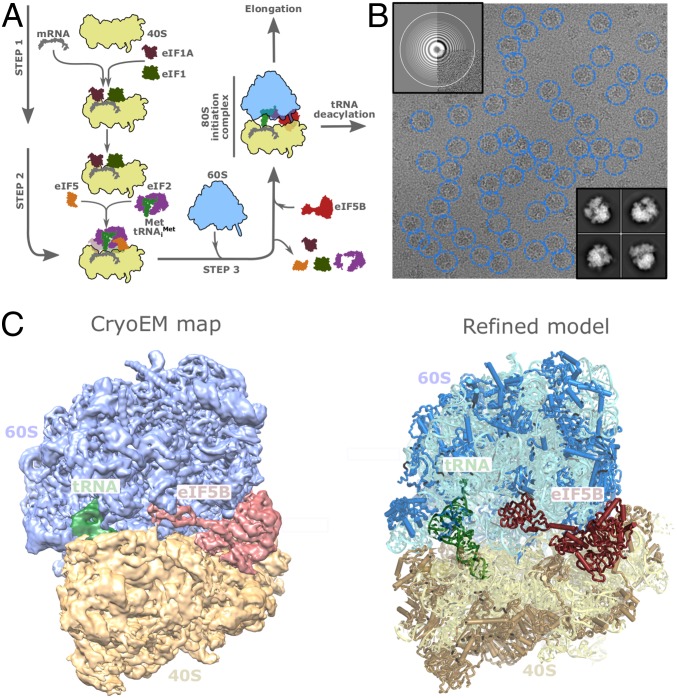
Ribosome-bound eIF5B structure. (*A*) Cartoon schematic showing the enzymatic preparation of the in vitro initiation reaction imaged by cryo-EM. (*B*) Representative electron cryomicroscopy micrograph showing selected 80S particles in blue circles. Examples of CTF spectrum of the micrograph (*Upper Left*) and 2D classes are shown (*Lower Right*). (*C*, *Left*) Electron-density map showing the reconstructed 80S/tRNA/eIF5B complex. The 60S large ribosomal subunit is colored in blue, the 40S small ribosomal subunit is colored in yellow, tRNA is in green, and eIF5B is in maroon. (*C*, *Right*) Refined model with components colored with the same color scheme.

We sought to assemble a late initiation complex with eIF5B stalled at the 80S ribosome by employing purified yeast components and mimicking an initiation reaction in vitro (Fig. 1*A*). A preincubation of 40S with initiation factors eIF1 and eIF1A and mRNA was followed by the addition of Met-tRNA_i_^Met^ as a TC with eIF2 and GTP. A final addition of eIF5B and 60S preincubated with the nonhydrolyzable GTP analog (GDPCP) effectively stalls the factor on the ribosome. As reported, eIF5B’s affinity for GTP/GDP is very similar, so no GTP-Exchanging Factor (GEF) is needed either in vitro or in vivo (33). After large cryo-EM dataset collection and extensive maximum likelihood classifications in RELION (34, 35), we identified a homogeneous class of particles where density for the expressed construct of eIF5B could be identified, reaching a global resolution of 3.6 Å (Fig. 1 and *SI Appendix*, Fig. S1 and Table S1).

**Fig. 2.**
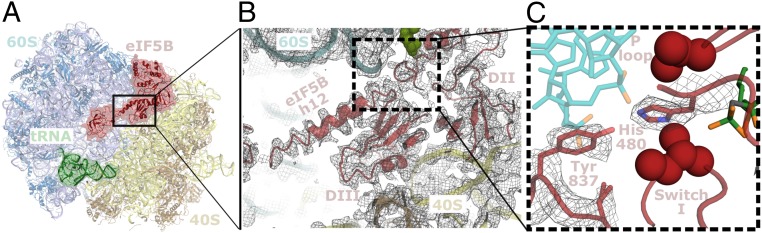
Intramolecular interactions of eIF5B in the ribosome-bound conformation. (*A*) Overall view of eIF5B (red), p/PE tRNA (green) bound to the intersubunit space of the ribosome between the 60S subunit (cyan) and the 40S subunit (yellow). (*B*) Zoomed-in view of eIF5B showing h12 contacting the G-domain and domain III (DIII) “clamped” between the 60S and the 40S. (*C*) Zoomed-in view of the area around tyrosine 837 of eIF5B in the vicinity of the γ-phosphate of the GTP analog molecule (green). Tyrosine 837 is oriented toward the catalytic histidine of eIF5B (His480) which is inserted through the “hydrophobic gate” formed by eIF5B residues Ile438 and Val414 (maroon spheres).

The reconstruction exhibits a slightly rotated configuration of the 40S (∼3°) and contains a tRNA in hybrid p/PE configuration (36), where the Anti-codon Stem Loop (ASL) of the tRNA is deeply inserted in the P site of the small subunit, and it is base-paired with the AUG codon. The acceptor stem is populating a space in between the space normally populated by P and E tRNAs in their canonical states (*SI Appendix*, Fig. S2*A*). The L1-stalk is displaced inward, stabilizing the tRNA in this hybrid configuration (37). Density for the 4 domains of eIF5B could be identified in the maps, with the G-domain anchored to the 60S, domain II anchored to the 40S, and the unit formed by domain III, h12, and domain IV projected toward the Peptidyl Transfer Center (PTC). This tRNA/eIF5B configuration is peculiar, as there are no direct tRNA/eIF5B contacts. The initial Met-tRNA_i_^Met^ added to the reaction has suffered a deacylation event after its delivery to the P site, and its acceptor stem has transition halfway toward the E site, where it has been stabilized by the L1 stalk (37). Unexpectedly, eIF5B has been trapped in its active conformation, even in the absence of contacts with Met-tRNA_i_^Met^. We reasoned this peculiar “dead end” intermediate of the initiation route has been stabilized due to differences in composition and buffer conditions used to mimic the in vitro initiation reaction from previously reported reactions (13). *Kluyveromyces lactis* subunits and eIF5B in a slightly acidic (pH 6) buffer background were used in this work. These conditions have been previously used for other 40S initiation complexes studied by cryo-EM (12, 38), but not for a late intermediate with eIF5B and 60S. In these conditions, a large subpopulation of particles contains eIF5B stabilized in the active conformation, allowing the visualization of this conformation at high resolution by cryo-EM. In our maps, side-chains for eIF5B could be confidently assigned, and the determinants of eIF5B/ribosome interaction could be unambiguously defined (Fig. 2). The global ribosome-bound eIF5B conformation is identical to our previous lower-resolution reconstruction; thus we used this high-resolution reconstruction as a proxy for a bona fide late initiation complex (21).

### eIF5B Domain III Plays a Direct Role in GTP Hydrolysis.

How the ribosome activates GTPases involved in translation is well understood (16, 25). The G-domains present in these GTPases protect the γ-phosphate of GTP via 2 structured loops, named switch 1 and 2. These loops form a “hydrophobic gate,” which prevents the access of a universally conserved catalytic histidine located at the P-loop from accessing the γ-phosphate of GTP. Upon 60S subunit recruitment, key elements of the 60S rRNA located at the SRL “push” the catalytic histidine through the hydrophobic gate and position the residue in a conformation that promotes hydrolysis. Thus, the bulk of GTP regulation in GTPases involved in translation is located at the G-domain.

eIF5B, in its active, ribosome-bound conformation, positions the unit formed by domain III, h12, and domain IV deep inside the intersubunit space (Fig. 2*A*). This disposition places domain III in a notable location, “sandwiched” between the SRL of the 60S and ribosomal protein uS12 and 18S rRNA helix h5 of the 40S (Fig. 2*B* and ref. 39). Domain III thus, effectively, bridges the large and the small subunits and offers additional anchoring points to the 60S to facilitate its recruitment.

**Fig. 3.**
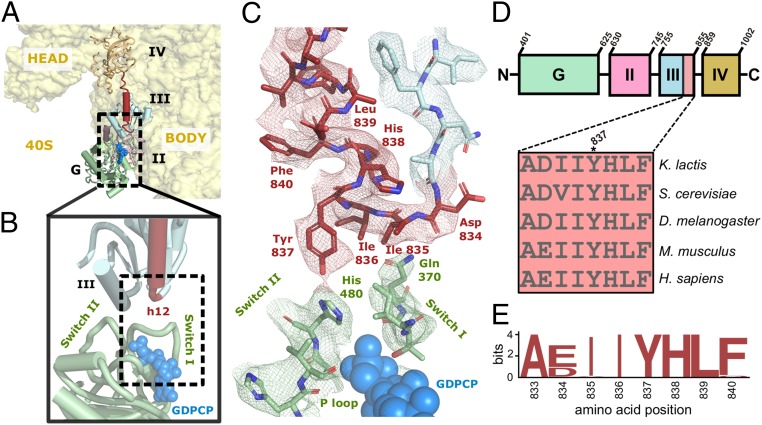
Interdomain interactions in the ribosome-bound conformation of eIF5B. (*A*) Domain distribution of eIF5B in its ribosome-bound conformation viewed form the top (60S not shown). eIF5B G-domain is colored green, domain II is pink, domain III is cyan, helix h12 is red, and domain IV is orange. (*B*) Detailed view of the interface of the G-domain with domain III around the GTP-binding region. (*C*) Experimental density colored according to the previously described color code for this area of eIF5B in the ribosome-bound conformation. Conserved residue Tyr837 is within interaction distance with the catalytic histidine of eIF5B (H480). Additionally, eIF5B residues flanking Tyr837 establish a network of interactions with other residues of the G-domain. (*D*) Conservation of the h12 N-terminal portion across representative eukaryotes. A schematic (*Top*) shows the domain organization of eIF5B, highlighting Tyr837 location. (*E*) Sequence logo plot illustrating the conservation of the protein sequence around Tyr837 on eIF5B homologs. The protein sequences of eIF5B across 54 species of eukaryotes were aligned with Clustal Omega and plotted as sequence logo plot.

The quality of our reconstruction allowed the unambiguous assignment of the side-chains for domain III of eIF5B (Figs. 2 *B* and *C* and 3*C*). In our refined model, we confirmed the position of a tyrosine residue at the base of h12 that is adjacent to domain III and within interacting distance with the catalytic histidine of eIF5B (Fig. 2*C*). A stacking interaction with the sugar of the 23S rRNA nucleotide 2997 aligns tyrosine 837 (*S. cerevisiae* numbering) toward the catalytic histidine, which is in its “active” configuration, inserted through the hydrophobic gate (25). This tyrosine residue was previously tentatively suggested to be relevant in GTP hydrolysis by eIF5B based on a low-resolution cryo-EM reconstruction (21) and given it is universally conserved in eukaryotes (Fig. 3 *D* and *E*). It has also been suggested to be relevant in bacterial (40) and mitochondrial initiation (41).

The interactions established by domain III with the 60S and the 40S also orient the domain in a specific direction relative to domain II (Fig. 3 *A* and *B*). In this orientation, a stretch of residues flanking tyrosine 837 seems to exert a relevant role in establishing interactions between the G-domain and domain III (Fig. 3 *A*–*C*). These residues are also located at the base of h12, the long α-helix–orienting domain IV to stabilize the Met-tRNA_i_^Met^ at the P site (20). We reasoned this stretch of residues, given its high conservation in eukaryotes (Fig. 3 *D* and *E*), could act as a signal integrator that communicates the presence of a properly delivered Met-tRNA_i_^Met^ at the P site to the G-domain of eIF5B and thus regulates GTP hydrolysis (9).

### Mutant eIF5B in Tyrosine 837 or Its Flanking Residues Is Defective In Vitro and In Vivo.

**Fig. 4.**
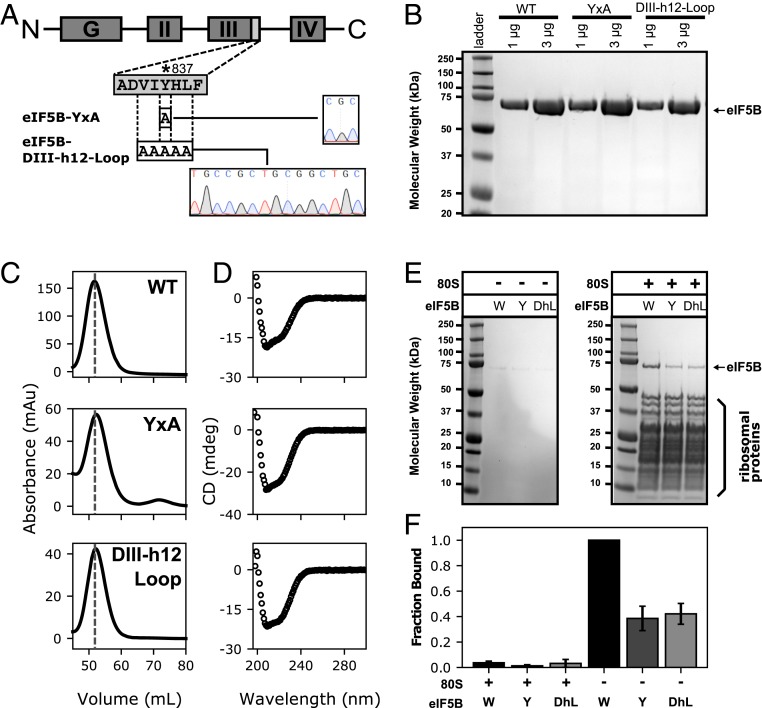
eIF5B mutant constructs, biophysical characterization, and 80S ribosome-binding assays. (*A*) Schematics showing eIF5B domain architecture, the stretch of residues where mutants are located as well as the engineered mutations with the corresponding sequencing chromatographs. (*B*) SDS/PAGE showing purified eIF5B wild type (WT) as well as the purified mutant proteins described in *A*. (*C*) Analytical gel-filtration chromatographs of the purified eIF5B and mutant proteins. The dashed lines are located at the same volume across all chromatographs. Similar hydrodynamic behavior indicates mutations do not affect biophysical properties of the mutants in the absence of ribosomes. (*D*) Circular dichroism spectrum showing the CD signature of eIF5B and mutants, confirming a similar content of secondary structure. (*E*) Ribosome-binding experiment of eIF5B and mutants. Shown, SDS/PAGE of the pellets over a 1M sucrose cushion. Significant decrease in ribosome binding could be observed for the mutant proteins in the same experimental conditions. (*F*) Quantification of ribosome-binding experiments showed in *E* with control experiments performed without 80S ribosomes on the left. The fractions were normalized in respect to the intensity of eIF5B WT pelleted in the presence of the 80S ribosomes. Error bars represent the SD from 3 experiments.

In order to test this hypothesis, we generated a set of mutants to test the in vitro and in vivo relevance of the residues identified in our high-resolution cryo-EM reconstruction (Fig. 4*A*). A point mutant substituting tyrosine 837 to alanine (eIF5B-YxA mutant) and a mutant additionally replacing the 2 residues flanking the tyrosine 837 at both sides to alanine (eIF5B-DIII-h12-Loop mutant) were created (Fig. 4*A*). Both mutant proteins can be produced recombinantly in *Escherichia* *coli* and can be purified following the same protocol as the wild-type protein (eIF5B-WT) with minor adjustment (Fig. 4*B*). The mutant proteins exhibit a hydrodynamic behavior in analytical gel filtration experiments similar to the WT protein, and their secondary structure contents are analogous to the WT as judged by circular dichroism (CD) spectrometry (Fig. 4*C*). We thus concluded the substitution of eIF5B tyrosine 837 for alanine, both in isolation or integrated in a stretch of 5 residues, does not perturb the folding or stability of the protein in isolation.

Next, we wanted to test the ability of the mutant proteins to engage ribosomes in a productive binary interaction. Purified 80S ribosomes, eIF5B, and nonhydrolyzable GTP analog were incubated and sedimented through a sucrose cushion. The sedimented ribosomal complexes were analyzed by SDS/PAGE and quantified. The results show the WT protein binds tightly to the 80S ribosomes in the presence of a nonhydrolyzable GTP analog, which is in excellent agreement with previously reported data (Fig. 4 *E* and *F* and ref. 42). Both mutants eIF5B-YxA and eIF5B-DIII-h12-Loop showed an approximately 2-fold reduction in ribosomal binding under the same experimental conditions (Fig. 4 *E* and *F*). In contrast to the WT protein, these results suggest a distorted ability of the mutant proteins to acquire an active, ribosome-bound conformation that is stable enough to be analyzed by pelleting assays (42).

**Fig. 5.**
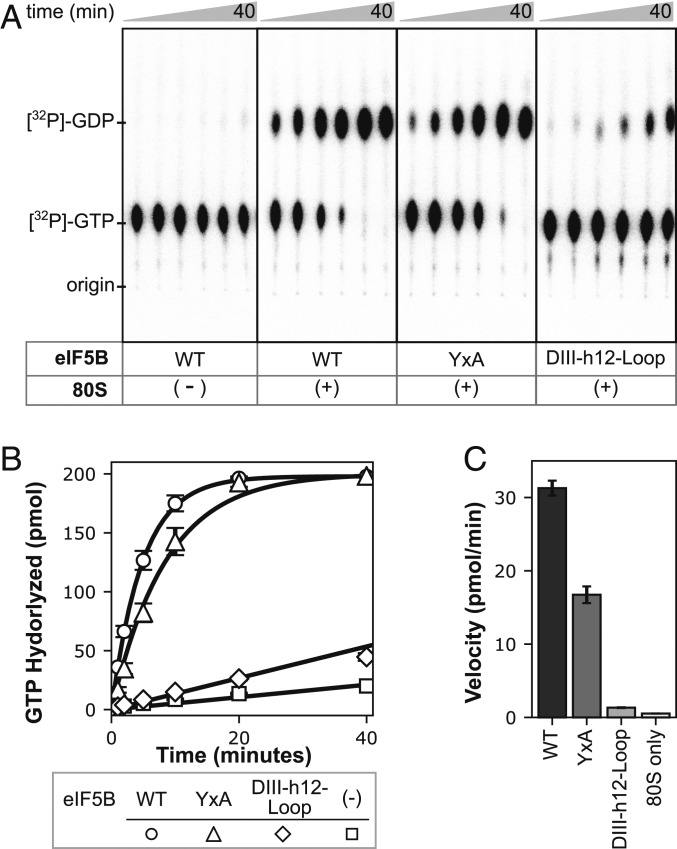
Ribosome-induced GTP hydrolysis activity of eIF5B and mutants. (*A*) Examples of radiographs of [^32^P]-GDP and [^32^P]-GTP separated from eIF5B GTP hydrolysis reactions using TLC. Each reaction contains 20 pmol of purified eIF5B proteins, 10 pmol of 80S ribosome (or no 80S for eIF5B-only reactions), and 200 pmol of GTP in 20 μL. Each lane is a time point taken and quenched from the reaction. Time points from 1 to 40 min are shown. (*B*) Quantification of GTP hydrolyzed by eIF5B proteins from TLC. The data points represent the average of 3 independent experiments (*n* = 3), and the error bars represent the SD of the experiments. The circle symbols represent the reaction with eIF5B-WT, the triangle symbols represent the reaction with eIF5B-YxA proteins, the diamond symbols represent the reaction with eIF5B-DIII-h12-Loop, and the square symbols represent the reaction with the 80S ribosome and no eIF5B. The linear lines represent linear fit of the corresponding dataset. The exponential lines (WT and YxA) represent single exponential fit of the datasets. (*C*) Quantification of the initial velocity of the reactions. The linear phase of the data was used to determine the initial velocity of each reaction. The error bars represent the SE of the fits.

We then assayed the in vitro, ribosome-dependent GTPase activity of eIF5B-WT, as well as the mutants, using a radiometric GTP hydrolysis assay. Briefly, we incubated eIF5B-WT and mutants with purified yeast 80S ribosomes in the presence of [γ-^32^P]-GTP and quenched the reaction at various time points (15). The products were separated into GTP and GDP using thin-layer chromatography (TLC) (Fig. 5*A*). The GTP-hydrolyzed were quantified and plotted against time (Fig. 5*B*). The eIF5B-WT protein hydrolyzed GTP at an initial rate of 31 pmol/min in the presence of 80S ribosomes (Fig. 5*C*). No significant GTP hydrolysis was observed in the absence of ribosomes (*SI Appendix*, Fig. S3). Mutant proteins exhibited a marked defect in ribosome-induced GTP hydrolysis. eIF5B-YxA exhibited a reduction of approximately half in the initial rate of GTP hydrolysis compared with the WT protein (16.7 pmol/min), whereas the eIF5B-DIII-h12-Loop mutant showed a dramatic 23-fold reduction (1.34 pmol/min, Fig. 5*C*).

With the previously described in vitro experiments, we demonstrated defects in both mutants in the 2 main functions of eIF5B, namely, ribosome binding and GTP hydrolysis. In order to relate these in vitro defects of the mutant proteins with defects in translation initiation in vivo, we made used of a previously established assay employing a yeast strain with a deletion in the gene *fun12* (*Δfun12*), which encodes the eIF5B protein (43). This deletion is not lethal in yeast, but generates a severe growth defect (44). Reintroducing the gene in *trans* from a vector to rescue the severe growth phenotype allows the in vivo testing of eIF5B mutants (42).

**Fig. 6.**
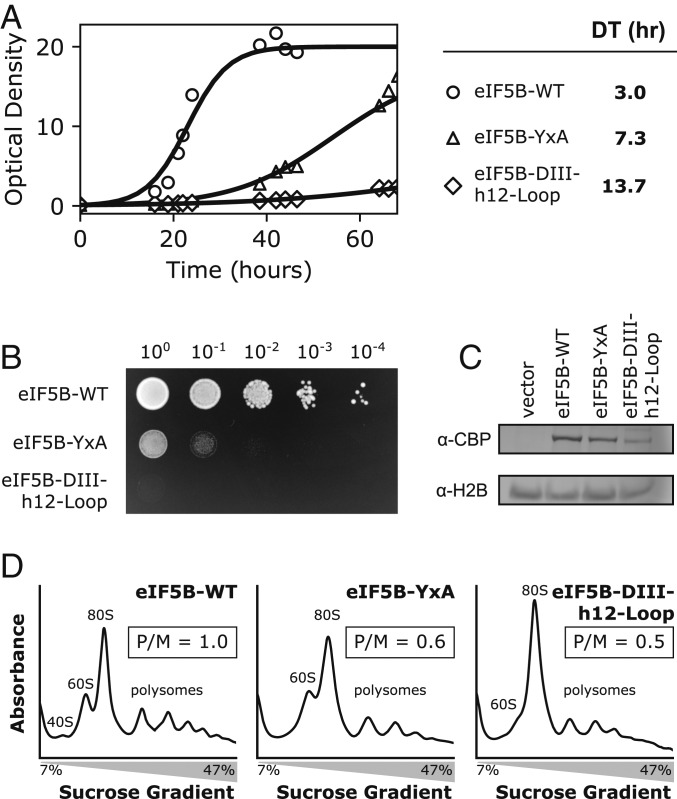
eIF5B Tyr837 mutants impact translation initiation in vivo. (*A*) A yeast strain lacking the *fun12* gene (*Δfun12* strain) was transformed with the pRS415 vector carrying nothing (vector control) or different eIF5B mutants under an inducible GAL promoter. On the *Left*, growth curves of yeast strains expressing the different eIF5B mutants in a *Δfun12* background in liquid cultures. On the *Right*, estimates of doubling time for each transformant. The recombinant expression of eIF5B-WT rescues the growth defect phenotype characteristic of the *Δfun12* strain (circles). eIF5B-YxA mutants are incapable of rescuing the defective phenotype (triangles). Additional mutation of flanking residues of Tyr837 yields an exacerbated toxic effect (diamond, mutant eIF5B-DIII-h12-Loop). (*B*) Solid culture experiments for the different transformants were grown on selection medium supplemented with 2% galactose to induce expression of the recombinant eIF5B proteins. Ten-fold serial dilutions were spotted on solid media and incubated for 6 d. In agreement with liquid culture experiments, eIF5B-YxA are unable to rescue the severe growth defect characteristic of the *Δfun12* yeast strain. The image shown is representative of 3 replicates. (*C*) Western blot analysis of eIF5B expression. Cell lysates prepared were subjected to immunoblot analysis using anti-CBD for eIF5B presence and antihistone H2B antibodies confirmed similar expression levels for all constructs. (*D*) Polysome profiles of whole-cell extracts of *Δfun12* yeast strain expressing eIF5B-WT (*Left*), eIF5B-YxA (*Middle*), and eIF5B-DIII-h12-Loop (*Right*). The WT protein exhibits a polysome/monosome ratio of 1, with a UV_260_ trace similar to previous studies (39). Expression of eIF5B-YxA induces a decrease in the amount of monosomes progressing into polysomes, reflected in a P/M ratio of 0.5. This behavior is exacerbated in yeast expressing the eIF5B-DIII-h12-Loop mutant (*Right*), where it can be observed a significant decrease in the number of polysomes with a concomitant accumulation of ribosomes in the monosome fraction (80S peak).

We thus introduced recombinant eIF5B by transforming the *Δfun12* strain with vectors carrying the eIF5B-WT gene or the mutant versions of it under an inducible galactose (GAL) promoter into strains with the *fun12* gene deleted. In this background, the *Δfun12* yeast strain transformed with an empty vector showed slow-growth phenotype with doubling time of 5.5 h, which is comparable with previously reported values of 5.1 h (14). The slow-growth phenotype is rescued by the recombinantly expressed eIF5B-WT to a doubling time of 3.0 h (Fig. 6*A*). The *Δfun12* yeasts expressing the eIF5B-YxA mutant grew at a doubling time of 7.3 h, while the *Δfun12* yeasts expressing the eIF5B-DIII-h12-Loop mutant showed the most severe growth defects, with doubling time of 13.7 h (Fig. 6*A*). Proper expression of protein products was confirmed by immunoblotting (Fig. 6*C*).

In parallel, we used a standard spot colony assay to evaluate the efficiency in slow-growth rescue of the mutant versions of eIF5B in solid culture (Fig. 6*B*). In agreement with the liquid culture doubling time estimations, the eIF5B-YxA mutant was unable to rescue the slow-growth defect phenotype, while the eIF5B-DIII-h12-Loop mutant exhibited an exacerbated slow-growth phenotype, which reflects an additional toxic effect of the mutant eIF5B-DIII-h12-Loop. A similar effect was observed for mutants of the catalytic histidine of eIF5B (39).

To examine the effect of these eIF5B mutations on general translation, we made use of polysome profile analysis (Fig. 6*D*). Yeasts transformed with eIF5B-WT exhibit a polysome profile in agreement with previously reported data (39) with a polysomes/monosomes ratio (P/M) of 1 (Fig. 6 *D*, *Left*). The eIF5B-YxA mutant protein exhibited moderate distortion in its ability to promote transition into elongation. Polysome profiles from yeasts transformed with this mutant exhibited an altered P/M ratio of 0.6 and a general decrease in the amount of polysomes (Fig. 6 *D*, *Middle*). However, in accordance with the in vitro data, the most prominent effect impacting the ability of monosomes to transit into polysomes was observed for the eIF5B-DIII-h12-Loop mutant (Fig. 6 *D*, *Right*). Polysome profiles corresponding to this mutant exhibited a significant accumulation of monosomes and a P/M ratio of 0.5. In agreement with the toxic effect impairing rescue of the severe growth phenotype both in liquid and solid culture, the expression of eIF5B-DIII-h12-Loop blocks the progression into elongation, “trapping” ribosomes in a late initiation state that is unable to assemble polysomes.

## Discussion

Ribosomes contain multiple binding sites intended to accommodate substrates of different natures: tRNAs, mRNAs, or protein factors (7). Communication and signal propagation between functional sites are essential for a correct, processive ribosome (16). A well-studied example of how distant ribosome-binding sites communicate is exemplified by the discrimination between cognate and noncognate codon/anticodon pairs during elongation (45). Other mechanisms, especially in the larger, more complex mammalian ribosomes, operate in a similar logic (4): multidomain protein factors recognize a specific state of the ribosome, and if certain criteria are met, a signal is propagated and amplified by the coupling of the ribosomal movements with conformational changes of the protein factor to regulate that particular pathway.

Initiation can be integrated in this paradigm (9, 46). Met-tRNA_i_^Met^ has to be delivered to the P site to recognize a particular AUG codon in the mRNA. A signal of proper delivery triggers the recruitment of the large ribosomal subunit, and protein synthesis transitions into elongation. The GTPase eIF5B is responsible for coordinating these events, integrating inputs of different natures in varying contexts.

As a classic GTPase, eIF5B is activated off the ribosome by GTP binding (15, 33). This event causes the transition from an autoinhibited, GDP-bound state to an activated, GTP-bound state. The active state involves the restructuring of the elements surrounding the GTP in the G-domain and the “release” of domains III and IV from the autoinhibited conformation in a mechanism termed “domain release” (19). However, a simple GTP-ON/GDP-OFF mechanism cannot comprehensively explain the multiple inputs eIF5B has to integrate in order to determine the final outcome: transition or not into elongation.

**Fig. 7.**
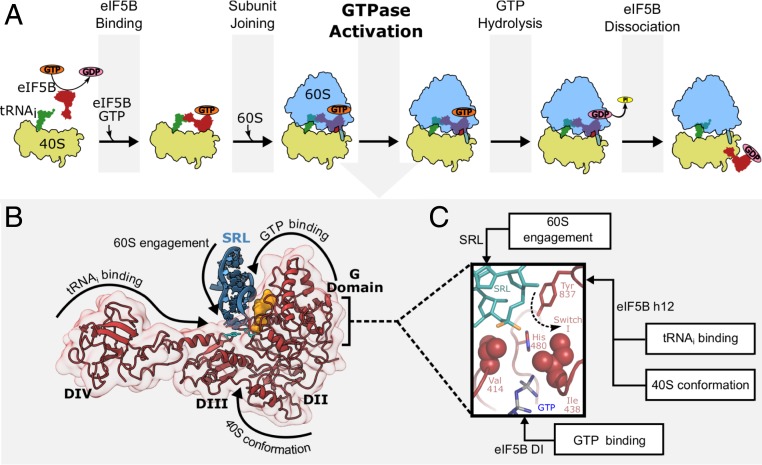
Presence of Met-tRNA_i_^Met^ in the P site is detected by eIF5B, and this signal is propagated to the GTPase center of the ribosome via interdomain communications. (*A*) Cartoon representation of the main molecular events proposed for eIF5B mode of action. From left to right: exchange of GDP (pink) with GTP (orange) allows the release from a rigid, self-inhibited configuration of eIF5B (red). This “domain release” allows binding to the small ribosomal subunit (40S, yellow) and initial recognition of Met-tRNA_i_^Met^ at the P site (green). Recruitment of the large ribosomal subunit (60S, blue) is then facilitated by eIF5B in its GTP-bound state. In the subsequent GTPase activation process, specific nucleotides of the 60S located at the SRL orient eIF5B catalytic histidine toward the γ-phosphate of GTP, which results in an active form of eIF5B. After GTP hydrolysis and phosphate release, conformational changes in eIF5B-GDP result in its dissociation from the ribosome. (*B*) eIF5B is composed of 4 domains, each of them performing specific tasks, recognizing specific inputs from several sources: domain IV recognizes a properly delivered Met-tRNA_i_^Met^ at the P site, domain III senses the conformational state of the 40S with respect to the 60S, domain II engages the 40S, and the G-domain harbors the bulk of the GDP/GTP regulation as well as the 60S-binding determinants. (*C*) We have located a stretch of residues in eIF5B, outside the G-domain, that is essential for the integration of the several inputs needed for GTP hydrolysis and progression into elongation. The center of this regulation is a universally conserved tyrosine which assists the catalytic histidine in activating eIF5B.

In order to allow progress into elongation, eIF5B is thus responsible for integrating inputs of very different natures, such as cellular GTP concentration, 40S conformation, Met-tRNA_i_^Met^ presence at the P site, and, finally, an appropriate recruitment of the 60S (Fig. 7*A*). Additionally, eIF5B has to operate in different ribosomal contexts as it initially binds the post-48S complex and after eIF2/eIF1 release and 60S recruitment, in a full (80S) ribosome context (14, 23). All this could only be accomplished through the multidomain nature of eIF5B, where specific domains “sense” specific inputs (Fig. 7*B*). Domain IV of eIF5B is projected deep into the intersubunit space to recognize a properly delivered Met-tRNA_i_^Met^ (22). This domain is also responsible for interaction with other important initiation factors like eIF1A and eIF5 (47). Domain III couples the rotational state of the 40S to the G-domain. Domain II contributes additional anchoring points to the 40S (39), and, finally, the G-domain is responsible for GTP binding and the bulk of interactions with the large subunit (21).

Recent real-time, single-molecule experiments have uncovered the dynamics of eukaryotic initiation, demonstrating significant differences with its prokaryotic counterpart (26, 48). Notably, a long residence time was observed for eIF5B on the 80S complex after 60S recruitment and prior to GTP hydrolysis (defined as the 80S-Initiation Complex, 80S-IC). A rearrangement of the 80S-IC during this eIF5B long residence time has been proposed to occur. With the ability of eIF5B in integrating multiple inputs through its multidomain architecture, we speculate that this rearrangement of the 80S-IC involves the positioning of the conserved tyrosine 837 toward the catalytic histidine of eIF5B, once all inputs are checked and “approved.” Thus, tyrosine 837 and its flanking residues are key in coordinating the different inputs needed to allow progression into elongation, further underscoring the importance of interdomain communications in eIF5B for proper discrimination of ribosomal states (Fig. 7*C*).

An additional role for eIF5B has been described, related to start-codon identification and AUG selection accuracy (28). In conjunction with eIF5, eIF1A, and the 40S subunit, eIF5B exerts a regulatory role in preventing the identification of the correct AUG codons, forcing the scanning machinery to assemble initiation complexes in upstream AUGs (uAUGs). Under stress conditions, where eIF2 is phosphorylated and is unable to deliver Met-tRNA_i_^Met^ to the P site of the 40S subunit, eIF5B is able to substitute eIF2 and operate the eukaryotic initiation machinery in a “bacterial-like” mode (29). As these events take place in the context of the 40S and involve mainly domain IV, we believe the mechanism described here for tyrosine 837 and its surrounding residues is likely not involved. However, given the critical role DIII of eIF5B plays in the interaction with the 40S, it is probably a prerequisite for a successful 60S recruitment a correct positioning of the tyrosine 837 and its flanking residues, which is influenced by the interactions established by the distant domain IV.

In summary, our results complement previous studies on eIF5B and represent a step forward in the understanding, within a structural framework, of how this key initiation factor functions. These operations involve tasks beyond large subunit recruitment, as it has been demonstrated that eIF5B exerts a critical role in initiation AUG codon selection accuracy or Met-tRNA_i_^Met^ delivery in noncanonical scenarios like viral infection or cellular stress (29, 49). How eIF5B is able to mediate these expanded roles will demand further studies.

## Materials and Methods

### In Vitro Initiation Reaction Components and Reaction Setup.

Ribosomal subunits were purified from *K. lactis* (strain GG799) and used in an in vitro initiation reaction as previously described for *S. cerevisiae* (21). Briefly, 80S ribosomes were pelleted through a sucrose cushion and purified from sucrose gradients consisting of 20 mM 2-(*N*-morpholino) ethanesulfonic acid (MES) pH 6, 50 mM KCl, 8 mM Mg-acetate, 2 mM DTT, and 10 to 40% sucrose. The 80S ribosomes were split into subunits by dialyzing against 20 mM MES pH 6, 600 mM KCl, 8 mM Mg-acetate, and 2 mM DTT and separated through sucrose gradients consisting of the same splitting buffer with 10 to 30% sucrose. The 40S and 60S ribosomal subunits were flash-frozen and stored in −80 °C. Unstructured mRNA with sequence 5′-GGAAUCUCUCUCUAUGCUCUCUCUC-3′ was synthesized by Integrated DNA Technology. Yeast Met-tRNA_i_^Met^ was purified as previously described (13). Initiation factors eIF1, eIF1A, eIF2, and eIF5 were purified from *S. cerevisiae* as previously described (13).

An N-terminally truncated eIF5B from *K. lactis* (residue 361–967, with a molecular weight of 67 kDa) was codon-optimized for *E. coli*, synthesized, and cloned in a T7-based expression vector that added a 6xHis-tag followed by a TEV protease cleavage site at the N-terminal. This construct is an equivalent of the truncated *S. cerevisiae* eIF5B residue 396–1002 used in previous studies (17, 21). Site-directed mutagenesis was performed on this construct to prepare eIF5B-YxA mutant where Tyr837 (Tyr802 in *K. lactis*) was substituted to alanine and eIF5B-DIII-h12-Loop mutant where residues 835–839 (800–804 in *K. lactis*) were substituted into alanine residues. Overexpression in *E. coli* strain BL21(DE3) (Invitrogen) was followed by chromatographic purification using HisTrap, Q-HP, and Sephacryl S-200 (GE Healthcare). The purified protein was concentrated and snap-frozen in liquid nitrogen.

The 80S initiation complex is enzymatically assembled using purified yeast protein components. Initially, 0.3 μM of the 40S subunits were mixed with excess of 3 μM mRNA, 0.9 μM Met-tRNA_i_^Met^, 0.3 μM eIF1, 0.3 μM eIF1A, and 10 μM GTP and incubated at 30 °C for 2 min. Subsequently, 0.3 μM eIF2 and 0.3 μM eIF5 were added and incubated for an additional 2 min. At the final step, 0.3 μM 60S subunit, 3 μM eIF5B, and 1 mM GDPCP (SIGMA) were added in the mixture and incubated for 2 min prior to chilling on ice.

### Electron Microscopy.

The 80S initiation complex was diluted to ∼80 nM, and 3 μL were spotted on glow-discharged holey carbon grids (Quantifoil R2/2) deposited with a homemade continuous thin carbon film (estimated to be ∼30 Å thick) for 30 s. Each grid was blotted for 2.5 s and flash-frozen in liquid ethane using an FEI Vitrobot. The grids were imaged with an FEI Polara G2 microscope operating at 300 kV. Defocus values in the final dataset ranged from 1.6 to 3.6 µm. Images were recorded manually on a back-thinned FEI Falcon III detector at a pixel size of 1.07 Å in linear mode. The individual frames from the detector (36 frames for each 1-s exposure) were captured and stored on disk. All electron micrographs were evaluated for astigmatism and drift.

### Image Processing.

CTF estimation was performed with GCTF (50), and automatic particle picking was done with Gautomatch without a specific template using a diameter of 280 pixels. All 2-dimensional (2D) and 3-dimensional (3D) refinements were performed using RELION (34). We used reference-free 2D class averaging to discard 60S subunits and defective particles, resulting in 64,815 particles of the final dataset for subsequent 3D refinement and classification. Refinement of all particles against a single model (a 60 Å low-pass-filtered version of EMDB-2275) yielded a preliminary, consensus reconstruction with local fuzzy density for the 40S subunit, the L1 stalk, and the factor. Subsequently, we employed local classification with masks covering the intersubunit space to identify a class of 29,712 particles, for which all 4 domains of the factor showed clear density. Reported resolutions are based on the gold-standard FSC = 0.143 criterion and involved only soft masking (51, 52). Prior to visualization, all density maps were corrected for the modulation transfer function (MTF) of the detector and then sharpened by applying a negative B− factor that was estimated using automated procedures. Manual rebuilding was done using COOT (53), and the final model was refined with REFMAC (54) following previously established protocols.

### GTP Hydrolysis Assay.

Reactions containing 10 pmol *K. lactis* 80S ribosomes, 20 pmol eIF5B, and 10 µM GTP (with 0.01 µCi [γ-32P] GTP) in 20 µL GTPase buffer (20 mM Hepes pH 7.4, 50 mM KCl, 2 mM DTT, 2.5 mM Mg-acetate) were incubated at 30 °C. Reactions were quenched by taking out 2 µL aliquots and mixed with 6 µL of 50 mM EDTA and 90% formamide. The products were analyzed by TLC on PEI-F cellulose plates (EMD chemicals) with 0.85 M potassium phosphate [pH 3.4] as the mobile phase. The TLC plates were exposed on a phosphorimager. The amount of GTP hydrolyzed were quantified with ImageQuant v5.2 and plotted as a function of time. The rates of GTP hydrolysis were determined by fitting with a single exponential function with the form *y* = A(1 − exp[−*k* * *x*]). The initial rate/velocity of the GTP hydrolysis were determined by fitting the linear phase of the reaction with *y* = *v* * *x*.

### CD Spectrometry.

eIF5B samples were diluted to final concentration of ∼0.35 mg/mL in a buffer containing 10 mM K_2_(PO_4_) and 300 mM KCl. CD spectra were taken with Chirascan V100 CD Spectrometer (Applied Photophysics) using a 0.5-mm quartz cuvette between 200 and 300 nm at a set temperature of 25 °C.

### Ribosome-Binding Assay.

Reactions containing 30 pmol eIF5B, 15 pmol *K. lactis* 80S ribosomes (or none for eIF5B-only reactions), and 0.1 mM GDPNP in 30 µL reaction buffer (20 mM Hepes pH 7.4, 50 mM KCl, 2 mM DTT, 2.5 mM Mg-acetate) were incubated at 30 °C for 5 min. The reactions were chilled on ice and layered on top of 200 µL of prechilled sucrose cushion (20 mM Hepes pH 7.4, 50 mM KCl, 1 M sucrose, 2 mM DTT, 10 mM Mg-acetate). The samples were centrifuged at 4 °C for 3 h at 80,000 rpm in a TLA100 rotor. The ribosome-bound fractions were sampled by dissolving the pellet in 20 µL of reaction buffer. The samples were separated by SDS/PAGE and stained with Coomassie brilliant blue. The band was quantified using Image Lab Software (Bio-Rad).

### Yeast Complementation and Growth Assays.

*S. cerevisiae* eIF5B knockout strains (Dharmacon) (MATa his3Δ1 leu2Δ0 met15Δ0 ura3Δ0) were transformed with pRS415 plasmid containing the CBD-tagged WT or mutant eIF5B genes from *S. cerevisiae* (equivalent to the *K. lactis* protein construct described in the previous sections with N-terminal truncation) under a galactose-inducible promoter. Transformants were streaked on selection medium. Single colonies were isolated and grown in liquid selection media supplemented with 2% raffinose at 30 °C. For spot assays, saturating cultures of yeast cells were diluted to 1 optical density (OD) in sterilized water. Four 10-fold serial dilutions were performed for each sample and spotted on a selection medium containing 2% galactose. The plates were incubated at 30 °C for 5 d and imaged. To determine the doubling time, liquid cultures were diluted to 0.1 OD with selection media supplemented with 1% galactose and grown in culture flasks at 30 °C. The optical-density measurements at various time points were plotted against time and fitted with a logistic growth model: *y* = K/(1 + [(K − 0.1)/0.1] * np.exp[−r * t]). The doubling times during exponential growth in liquid medium were reported (44). The expression of recombinant eIF5B is determined by immunoblot. Briefly, harvested yeast pellets were lysed with glass beads and span down at 14,000 rpm for 10 min. The total protein concentration of the cell lysate was determined by BCA assay and ran on an SDS/PAGE gel. The separated proteins bands were transferred to a 0.2-μm nitrocellulose membrane and blotted with primary antibodies against CBP (Genscript) or Histone H2B (Active Motif), followed by blotting with Alexa Fluor 488 conjugated secondary antibody (ThermoFisher) prior to imaging.

### Polysome Analysis.

Polysomes were analyzed by sedimentation in sucrose gradients using a previously described protocol with slight modifications (39, 42). Briefly, yeast cells were grown in selection media containing 1% galactose to 1 OD as described in the previous section. The cultures were incubated with 50 μg/mL of cycloheximide for 10 min at 30 °C and collected in centrifuge bottles containing crushed ice. The cell pellets were resuspended in lysis buffer containing 20 mM Tris⋅HCl, pH 7.5, 50 mM KCl, 10 mM MgCl_2_, 2 mM DTT and lysed by vortexing with glass beads for 1 min and incubating on ice for 1 min, for a total of 5 cycles. The lysates were span at 14,000 rpm for 10 min at 4 °C. The OD of the supernatant was measured by a UV-Vis Spectrophotometer at 260 nm, and 6 OD of each sample were loaded to Beckman SW40 7 to 47% sucrose gradients. The samples were centrifugated at 39,000 rpm for 2 h at 4 °C and analyzed with a gradient analyzer equipped with UV detector set at 260 nm. A Svitzky-Golay filter was applied to remove the noises from the detector.

### Data availability.

Atomic coordinates have been deposited in the RSCB PDB with accession code 6UZ7 and cryo-EM maps have been deposited in the EMDB with accession code EMD-20952.

## Supplementary Material

Supplementary File
