# Supplementary material for: Long-range interdomain communications in eIF5B regulate GTP hydrolysis and translation initiation
Source: Proc Natl Acad Sci U S A. 2020 Jan 3;117(3):1429–37. doi: 10.1073/pnas.1916436117 (PMC6983393; doi:10.1073/pnas.1916436117)
Supplement: Supplementary File [file pnas.1916436117.sapp.pdf]

PNAS
www.pnas.org

Supplementary Information for

Long range inter-domain communications in eIF5B regulate GTP hydrolysis and translation initiation.

Bridget Y. Huang and Israel S. Fernández

Israel S. Fernández

Email: isf2106@cumc.columbia.edu

This PDF file includes:

- Supplementary text
- Figures S1 to S3
- Tables S1
- References for SI

www.pnas.org/cgi/doi/10.1073/pnas.1916436117

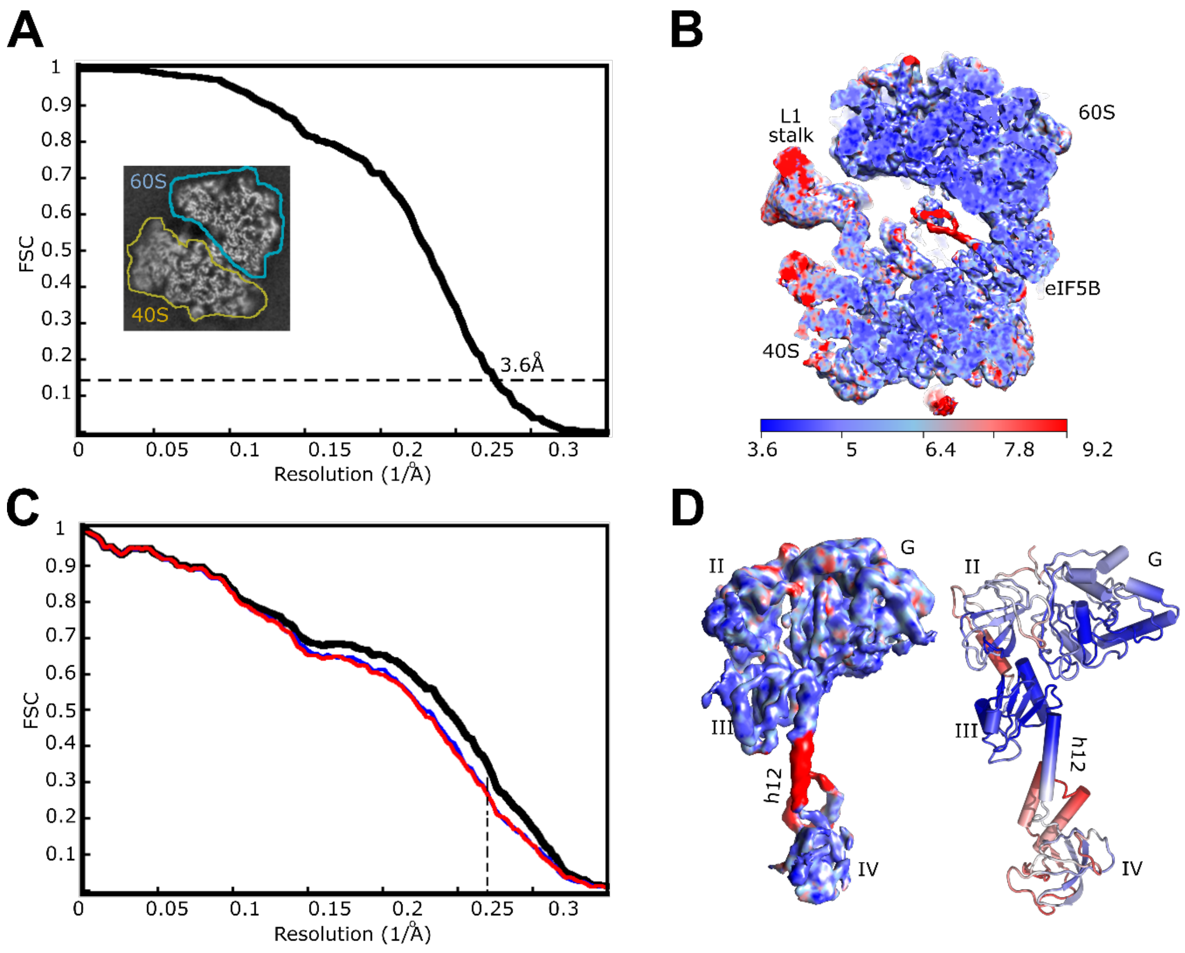

Fig. S1. Fourier Shell Correlation curves and local resolution. (*A*) Fourier Shell Correlation (FSC) computed from the two half maps of the final subset of particles after classification. The resolution is estimated to be 3.6Å using the 0.143 criterion (1). Inset, slice-through of the final map showing the high local resolution for both ribosomal subunits except for the tip of the head of the 40S, which appears to be more mobile. (*B*) Slice-through of the final, unsharpened map colored according to the local resolution as reported by RESMAP (2). Resolution beyond 4 Å can be observed for the 60S and 40S as well as for domains G, II and III of eIF5B. More flexible areas like the L1-stalk, the tip of the head of the 40S, and domain IV of eIF5B are observed at lower resolution. (*C*) Map-versus-model cross validation FSC. The final model was validated using

standard procedures. FSC of the refined model against half map 1 (blue) overlaps with the FSC against half map 2 (red, not included in the refinement). The black curve corresponds to the FSC of the final model against the final map. (*D*) Left, unsharpened density for eIF5B colored according to local resolution using the same scale as in B. Right, final model for eIF5B colored according to the refined atomic B-factors computed by REFMAC (3) (blue low, red high).

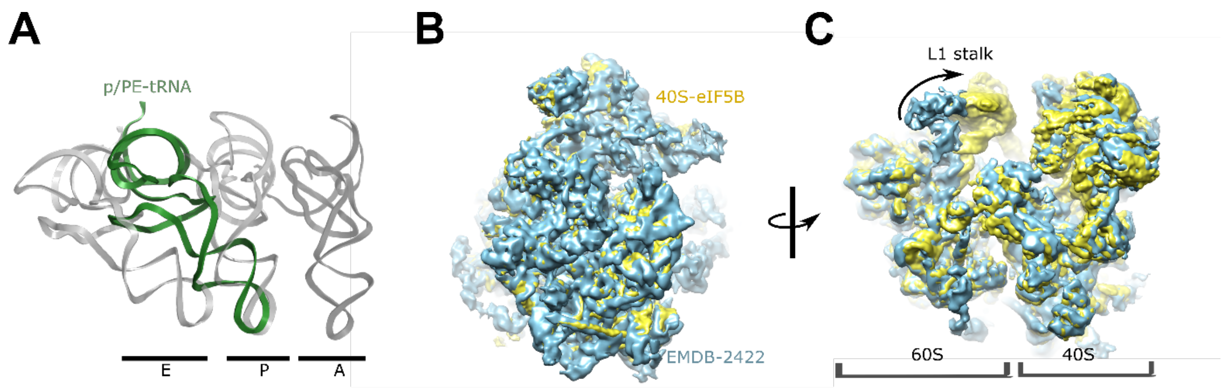

Fig. S2. tRNA conformation and superposition with low resolution map. (*A*) Final refined model of the tRNA in the present reconstruction (green) superposed with canonical tRNAs in grey (PDB-ID 4V5C). The anticodon stem-loop is located in the P site of the 40S, whereas the CCA end is placed in between the P and E sites of the 60S (p/PE configuration). (*B*) Superposition of our previous lower-resolution reconstruction (cyan, EMDB- 2422) with the present one (yellow), showing the similar degree of 40S rotation. (*C*) A 90 degree rotated view from the position showed in B. The arrow highlights an inward displacement of the L1-stalk in the current structure that stabilize the tRNA.

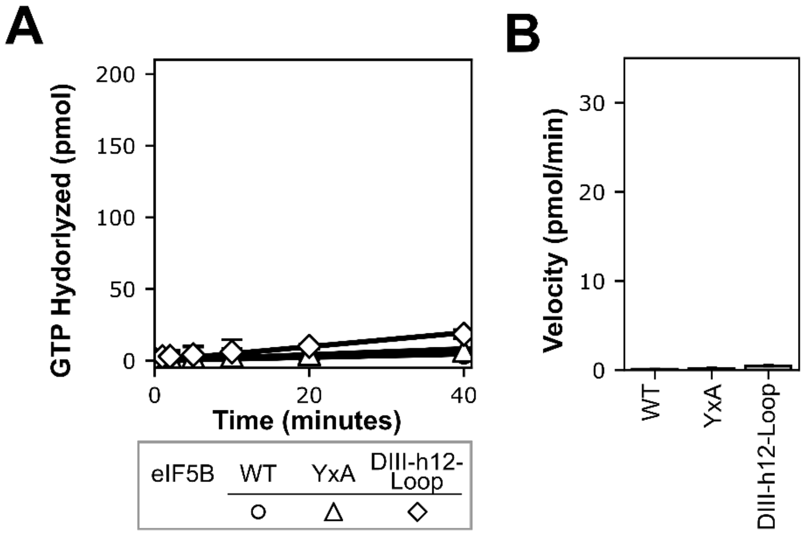

Fig. S3. GTP hydrolysis by eIF5B is residual in the absence of ribosomes. (A) Radiometric assay performed in the absence of ribosomes, showing evidence of negligible values of GTP hydrolysis. (*B*) Quantification of data presented in (*A*).

Table S1

Cryo-EM data collection, refinement and validation statistics

**Data collection and processing**	
Magnification	96,000
Voltage (kV)	300
Electron exposure (e–/Å^2)	70.91
Defocus range (μm)	-1.6/-3.6
Pixel size (Å)	1.07
Symmetry imposed	C1
Initial particle images (no.)	64,815
Final particle images (no.)	29,712
Map resolution (Å)	3.6
FSC threshold	0.143
Map resolution range (Å)	3-8
**Refinement**	
Initial model used (PDB code)	3U5B
Model resolution (Å)	3.6
FSC threshold	0.5
Model resolution range (Å)	3.6-8
Map sharpening *B* factor (Å^2)	-86.71
Model composition	
Non-hydrogen atoms	211,193
Protein residues	95,971
Ligands	32
B factors (Å^2)	110.6
R.m.s. deviations	
Bond lengths (Å)	0.0072
Bond angles (°)	1.04
Validation	
MolProbity score	2.12
Clashscore	1.81
Poor rotamers (%)	4.82%
Ramachandran plot	
Favored (%)	85.15
Allowed (%)	89.97
Disallowed (%)	3.98

References.

1. P. B. Rosenthal, R. Henderson, Optimal determination of particle orientation, absolute hand, and contrast loss in single-particle electron cryomicroscopy. *J Mol Biol* **333**, 721-745 (2003).
2. A. Kucukelbir, F. J. Sigworth, H. D. Tagare, Quantifying the local resolution of cryo-EM density maps. *Nat Methods* **11**, 63-65 (2014).
3. G. N. Murshudov, A. A. Vagin, E. J. Dodson, Refinement of macromolecular structures by the maximum-likelihood method. *Acta Crystallogr D Biol Crystallogr* **53**, 240-255 (1997).
